# Size-Dependent Spontaneous Separation of Colloidal Particles in Sub-Microliter Suspension by Cations

**DOI:** 10.3390/ijms23158055

**Published:** 2022-07-22

**Authors:** Shiqi Sheng, Haijun Yang, Yongshun Song, Ruoyang Chen, Shanshan Liang, Haiping Fang

**Affiliations:** 1 School of Physics, East China University of Science and Technology, Shanghai 200237, China; shengshiqi@ecust.edu.cn (S.S.); songyongshun11@mails.ucas.ac.cn (Y.S.); ruoyang.chen@ecust.edu.cn (R.C.); liangshanshan@ecust.edu.cn (S.L.); 2 Interdisciplinary Research Center, Shanghai Synchrotron Radiation Facility, Zhangjiang Laboratory (SSRF, ZJLab), Shanghai Advanced Research Institute, Chinese Academy of Sciences, Shanghai 201204, China; yanghaijun@sinap.ac.cn; 3 CAS Key Laboratory of Interfacial Physics and Technology, Shanghai Institute of Applied Physics, Chinese Academy of Sciences, Shanghai 201800, China; 4 Wenzhou Institute, University of Chinese Academy of Sciences, Wenzhou 325001, China

**Keywords:** size-dependent separation, large separation distance, sub-microliter suspension, hydrated cation-π interaction

## Abstract

Great efforts have been made to separate micro/nanoparticles in small-volume specimens, but it is a challenge to achieve the simple, maneuverable and low-cost separation of sub-microliter suspension with large separation distances. By simply adding trace amounts of cations (Mg^2+^/Ca^2+^/Na^+^), we experimentally achieved the size-dependent spontaneous separation of colloidal particles in an evaporating droplet with a volume down to 0.2 μL. The separation distance was at a millimeter level, benefiting the subsequent processing of the specimen. Within only three separating cycles, the mass ratio between particles with diameters of 1.0 μm and 0.1 μm can be effectively increased to 13 times of its initial value. A theoretical analysis indicates that this spontaneous separation is attributed to the size-dependent adsorption between the colloidal particles and the aromatic substrate due to the strong hydrated cation-π interactions.

## 1. Introduction

Separating and isolating micro/nanoparticles in suspension, especially in small-volume specimens, is a critical step in various environmental and biomedical applications [1,2,3,4,5,6,7,8,9,10,11,12,13]. In order to handle precious and vital specimens of small volume, methods relying on precisely fabricated instruments and skilled operations have been developed over the last few decades, such as surface acoustic waves [14,15,16], magnetic control of paramagnetic/diamagnetic particles [17,18,19,20,21], dielectrophoresis [22,23] and microfluidic techniques [11,24,25,26]. However, separating small-volume specimens in an economical and widely applicable manner remains a great challenge [27], where a simple, maneuverable method with extremely low specimen consumption is the key prerequisite.

The coffee ring effect (CRE), which applies to small droplets, is a good candidate for the separation of small-volume specimens. As a sessile droplet evaporates, an outward capillary flow is generated due to the much faster evaporation rate near the three-phase contact line (TCL) [28,29,30,31]. Driven by this capillary flow, particles and biological entities of different sizes are transported to and captured at distinct positions within the narrow TCL region, where their diameters precisely match the thickness of the local liquid meniscus [32,33,34,35,36,37]. Unfortunately, the fairly narrow TCL region of the droplet leads to very short separation distances (e.g., several to tens of micrometers between particles with diameters of 40 nm and 1.0 μm) [32,34,37], which decreases as the contact angle of the surface increases [38]. Although Bansal et al. found that large particles (~0.9 μm in diameter) were uniformly distributed, while small particles formed a ring on hydrophobic substrates with the contact angle >95° (i.e., polydimethylsiloxane and gas diffusion layer) [39], most researches indicate that this CRE-based separation method can only apply to suspensions with very low specimen fractions (<0.04 vol.%) [32,33] on sufficiently hydrophilic substrates [32,33,34,35,36]. Our previous work demonstrated that the CRE can be effectively controlled by simply adding trace amounts of salt to colloid suspensions [40,41]. It can be contributed to the enhanced adsorption between the particles and the aromatic substrate through strong hydrated cation-π interactions [42,43,44,45]. Theoretically, this cationic control method is independent of the shape of TCL so that it should exclude the limitations imposed by the narrow TCL region for particle separation within a sessile droplet.

In this work, we experimentally achieve the spontaneous separation of fluorescent polystyrene particles by simply adding trace amounts of salt (i.e., MgCl_2_, CaCl_2_ or NaCl) in a suspension droplet with a volume down to 0.2 μL. The separation distance observed is at a millimeter level, which even enabled us to manually sample the deposit pattern. A theoretical analysis indicates that this spontaneous separation is attributed to the size-dependent adsorption between the colloidal particles and the aromatic substrate due to the strong hydrated cation-π interactions. These findings have direct implications for the development of simple, maneuverable and low-cost technologies for low-volume sample preparation.

## 2. Results and Discussion

**Figure 1:**
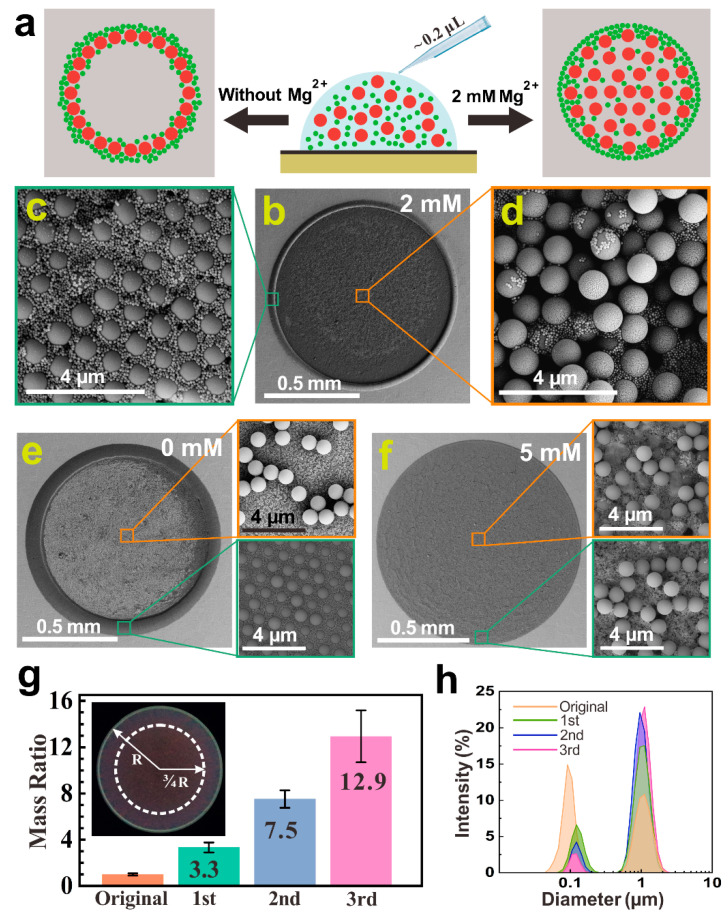
(**a**) Schematic diagram of the spontaneous separation between large particles (red spheres) and small particles (green spheres) in a droplet (light blue hemisphere) by adding trace amounts of cations. (**b**–**d**) SEM images of the deposit pattern dried from the suspension droplet containing bi-dispersed F-PS particles (1.0 μm and 0.1 μm in diameters) and 2.0 mM MgCl_2_ on graphene. (**e**,**f**) SEM images of the deposit patterns dried from the suspension droplets containing bi-dispersed F-PS particles without salts (**e**) and with 5.0 mM MgCl_2_ (**f**) on graphene. (**g**) The mass ratio between particles with diameters of 1.0 μm and 0.1 μm after multiple separating cycles in the presence of 2.0 mM MgCl_2_. The inset displays the selected sampling regions (i.e., the area surrounded by a white dashed circle) in the measurements. Error bars represent the standard deviation from at least three independent replicates. (**h**) Mass distribution with respect to particle diameters after multiple separating cycles. The intensity (%) represents the mass percentage of particles of different sizes.

In the experiment, aqueous suspensions of fluorescent polystyrene (F-PS) microspheres were used as reported previously [28]. The suspensions containing mono-dispersed F-PS particles with diameters of 1.0 μm and 0.1 μm (~1.0% *w*/*v*) were mixed in equal volumes to obtain a suspension containing bi-dispersed F-PS particles, followed by thoroughly mixing with MgCl_2_ solutions of different concentrations. Individual small droplets of these suspensions (0.2~1.5 μL) were then placed on a graphene substrate (Figure 1a). After evaporating at a temperature of 18 ± 3.5 °C and a relative humidity of 47 ± 3.0%, the morphologies of the dried deposits were recorded using scanning electron microscopy (SEM). Figure 1b–d shows the deposit pattern on a graphene substrate from a droplet of the suspension containing bi-dispersed F-PS particles and 2.0 mM MgCl_2_. We observed that large particles, with a diameter of 1.0 μm, were uniformly distributed throughout the deposit pattern while most of the small particles with a diameter of 0.1 μm were accumulated at the rim, clearly displaying the spontaneous separation of particles of different sizes. It should be pointed out that the width of the rim, where the majority of the small particles accumulated, was about only 1/5 of the radius of the pan-like deposit pattern (~0.5 mm as shown in Figure S2), suggesting a large separation distance between large and small particles, which benefits subsequent processing such as manually sampling from the deposit pattern and performing multiple separating cycles. When the MgCl_2_ concentration reached 5.0 mM, all the particles were uniformly distributed throughout the discoidal deposit pattern (Figure 1f). In contrast, for the salt-free suspension, all the particles were mixed together and accumulated at the rim of the deposit pattern, displaying a clear CRE (Figure 1e).

To demonstrate the capability of specimen post-processing of this cation-controlled method, we manually sampled the deposit patterns and measured the separation rate of particles after each separating cycle. The separation rate is denoted by the mass ratio r_m_ = M_1.0_/M_0.1_, where M_1.0_ and M_0.1_ are the total masses of F-PS particles with diameters of 1.0 μm and 0.1 μm in the sampling region (inset of Figure 1g), respectively. As shown in Figure 1g,h, the separation rate at the center of the deposit pattern increases from an initial value of 1.0±0.1 to 3.3±0.4 after the first cycle, and further extends to 12.9±2.2 after the third cycle, showing an enhanced separation ratio with multiple separating cycles.

**Figure 2:**
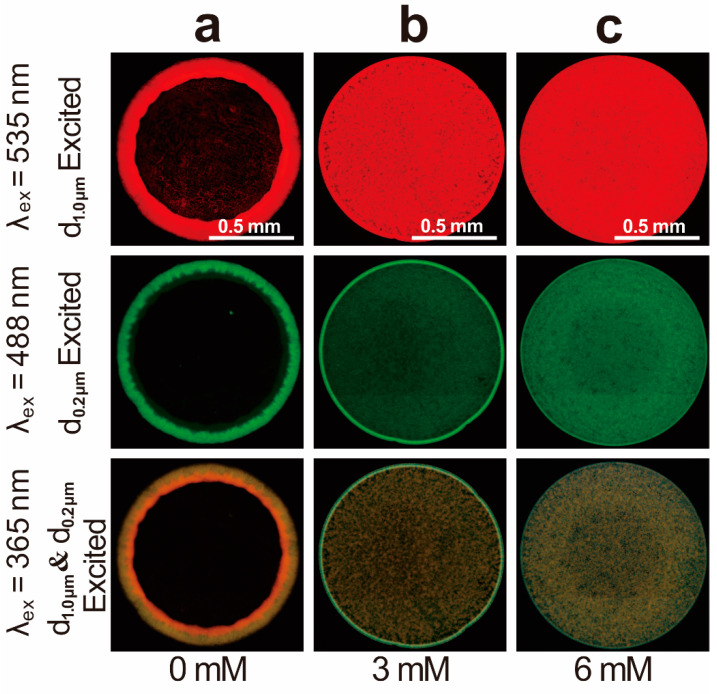
Fluorescence images of the deposit patterns dried from suspension droplets containing bi-dispersed F-PS particles (1.0 μm and 0.2 μm in diameters) and trace amounts of MgCl_2_ on PET substrate. Concentrations of MgCl_2_: (**a**) 0 mM, (**b**) 3.0 mM and (**c**) 6.0 mM. Each column shows the images of the same deposit pattern under excited lights of different wavelengths. At λ_ex_ = 535 nm (the first row), only the F-PS particles with a diameter of 1.0 μm are observed with red color; At λ_ex_ = 488 nm (the second row), only the F-PS particles with a diameter of 0.2 μm are observed with green color; At λ_ex_ = 365 nm (the third row), the F-PS particles with diameters of 1.0 μm and 0.2 μm are simultaneously observed with orange color and green color, respectively.

This cation-controlled spontaneous separation is also effective for particles with small size differences (e.g., d_large_/d_small_ = 5) and on other aromatic substrates such as the most common thermoplastic polymer resin of polyethylene terephthalate (PET). Figure 2 shows the fluorescence images of the deposit patterns dried from the suspensions containing bi-dispersed F-PS particles (1.0 μm and 0.2 μm in diameters) and different concentrations of MgCl_2_ on PET substrate. The red color traces the 1.0 μm diameter particles under excitation wavelength λ_ex_ = 535 nm (or orange color under λ_ex_ = 365 nm), while the green color traces the 0.2 μm diameter particles under λ_ex_ = 488 nm and λ_ex_ = 365 nm. For the MgCl_2_ concentration of 3.0 mM, a red pan-like pattern is observed under λ_ex_ = 535 nm (Figure 2b), indicating that the large particles uniformly distribute throughout the deposit. Meanwhile, a green ring pattern is observed under λ_ex_ = 488 nm, indicating that the small particles mainly accumulate at the rim of the deposit. The distinct distributions of particles with different sizes demonstrate that the spontaneous separation of particles is achieved by 3.0 mM MgCl_2_ on PET substrate. In contrast, the separation of particles is undetectable in the absence of salt (Figure 2a) or with an MgCl_2_ concentration of 6.0 mM (Figure 2c), consistent with the results in Figure 1.

**Figure 3:**
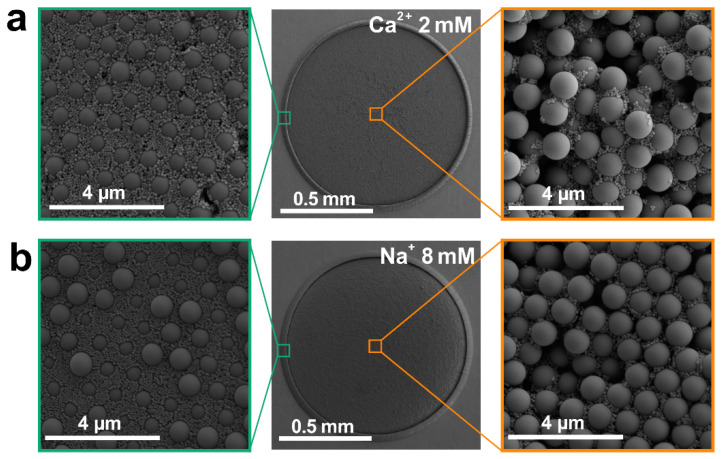
SEM images of the deposit patterns dried from suspensions containing bi-dispersed F-PS particles (1.0 μm and 0.1 μm in diameters) and different salts on graphene: (**a**) CaCl_2_ at 2.0 mM; (**b**) NaCl at 8.0 mM. The left and right show zoomed-in SEM images of selected areas at the rim and center, respectively.

The size-dependent spontaneous separation of particles is also observed for other salts such as CaCl_2_ and NaCl (Figure 3). By adjusting the salt concentration, the distribution of large and small particles within the deposit can be well controlled (Figures S3–S5). Interestingly, the optimum concentration to achieve effective separation between particles (1.0 μm and 0.1 μm in diameters) is about 8.0 mM for Na^+^ (Figure 3b and Figure S5), while it is about 2.0 mM for Ca^2+^ (Figure 3a and Figure S4) and Mg^2+^ (Figure 1b and Figure S3) on a graphene substrate, following the same strength order of their hydrated cation-π interactions [46]. These results indicate that a broad class of metal cations could be utilized to manipulate particle separations, which will benefit various practical applications with different specimen requirements.

Interestingly, the particle size ratio and the particle mass ratio, which are two important parameters affecting the self-assembly process for binary colloidal particle mixtures [47,48,49,50], only slightly affect this cation-controlled spontaneous separation. For example, the separation difference between the suspensions with the particle size ratio of d_large_/d_small_ = 10 and 5 is undetectable, as shown in Figure 1 and Figure 2. Moreover, the initial particle mass ratio in the suspension to be separated increases from 1.0 in the first cycle to 7.5 in the third cycle which only leads to a slight decrease in the separation rate from 3.3 to 1.7 (Figure 1g). These results indicate that there might be other mechanisms contributing to the cation-controlled separation.

**Figure 4:**
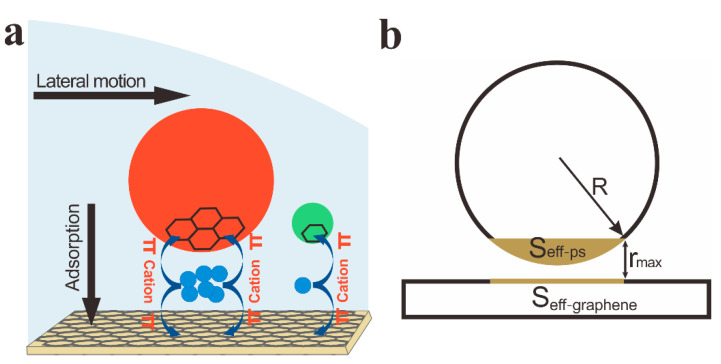
(**a**) Schematic diagram of cation-controlled separation. Cations mediate the adsorption between particles of different sizes and the aromatic substrate via hydrated cation-π interactions in a sessile droplet. The red/green and blue spheres denote the large/small colloidal particles and the cations, respectively. (**b**) Schematic diagram of the effect interaction areas of the hydrated cation-π interactions on the particle surface S_eff-ps_ and graphene substrate S_eff-graphene_.

Now we explore the physics underlying the size-dependent separation of particles by cations. As a sessile droplet of colloidal suspension with salts evaporating on an aromatic substrate, the lateral driving force $F_{L}$ imposed on an F-PS particle by the outward capillary flow due to the CRE is proportional to the product of flow velocity v and particle diameter **R**, according to Stokes’s Law (Figure 4a). Meanwhile, an attractive force **F_A_** acting on a particle close to the substrate is generated due to the cation-mediated hydrated cation-π interactions between the particle and the substrate. Obviously, the adsorption between a particle and the substrate increases with the cation concentration, resulting in a gradually suppressed CRE as the cation concentration increases [40]. For a given cation concentration, the adsorption probability of a cation onto an aromatic surface through hydrated cation-π interactions is proportional to the interaction area (i.e., the effective interaction areas of the F-PS particle S_eff-ps_ and the graphene substrate S_eff-graphene_ in Figure 4b). It is easy to verify that the attractive force **F_A_** acting on each F-PS particle close to the substrate is approximately proportional to the squared particle diameter **R^2^**, given the maximum interaction distance of hydrated cation-π interaction $r_{max}$ is small relative to R (Supplementary section PS4). As the particle diameter increases, the attraction of the aromatic substrate grows much faster than the lateral motion. Thus, the distributions of particles with different sizes in the deposit can be precisely tuned by adjusting the cation concentration. When the cation concentration is appropriate, large particles tend to be adsorbed onto the substrate, generating a uniform pattern after evaporation, while the majority of the small particles prefer to accumulate at the TCL, forming a ring pattern, so that separation is achieved (Figure 1a).

**Figure 5:**
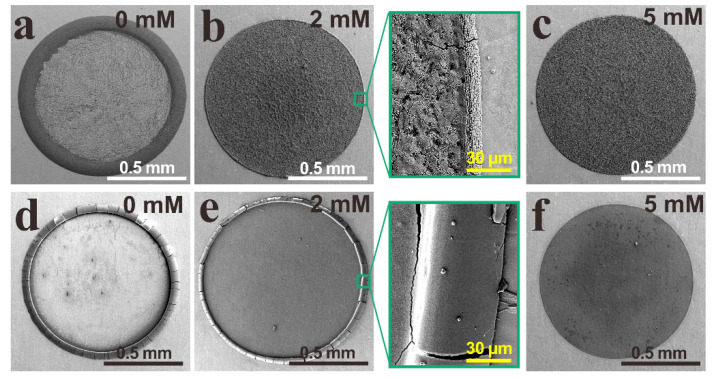
SEM images of the deposit patterns dried from suspension droplets containing mono-dispersed F-PS particles with different sizes and trace amounts of MgCl_2_ on graphene. The diameter of mono-dispersed particles: (**a**–**c**) 1.0 μm; (**d**–**f**) 0.1 μm. The zoomed-in SEM images show the selected areas at the rim of the corresponding deposit patterns.

To verify the mechanism of this cation-controlled separation, we further performed experiments with two series of suspensions containing mono-dispersed F-PS particles (1.0 μm or 0.1 μm in diameter) and different concentrations of MgCl_2_. For the deposit patterns dried from suspensions without cations (Figure 5a,d), we observed ring-like patterns with dark rims and blank centers, which display clear CRE. As the cation concentration increases, the grayscale difference between the rim and center of the deposit pattern gradually decreases for both of the suspensions containing mono-dispersed particles with different sizes, indicating that the CRE is suppressed gradually. Remarkably, the concentrations of MgCl_2_ for uniformly depositing particles (i.e., the pan-like pattern when the CRE is completely suppressed) are 2.0 mM and 5.0 mM for particles with diameters of 1.0 μm (Figure 5b) and 0.1 μm (Figure 5e), respectively. These results are consistent with our theoretical analysis that large particles are more easily adsorbed onto the substrate by cations, confirming the proposed cation-controlled mechanism for the size-dependent separation of particles.

## 3. Materials and Methods

### 3.1. Materials

Milli-Q water (Milli-Q, Millipore, 18.2 MΩ∙cm resistivity) was used for all experiments. The mono-dispersed fluorescent polystyrene (F-PS) particle suspensions were purchased from ACME microspheres, Inc. The nominated parameters are listed as follow: (I) 1.0 μm in diameter, 1% *w*/*v*, red fluorescent with excitation wavelength λ_ex_ = 535 nm and emission wavelength λ_ex_ = 610 nm; (II) 0.1 μm in diameter, 1% *w*/*v*, green fluorescent with excitation wavelength λ_ex_ = 488 nm and emission wavelength λ_ex_ = 525 nm; (III) 0.2 μm in diameter, 1% *w*/*v*, green fluorescent with excitation wavelength λ_ex_ = 488 nm and emission wavelength λ_ex_ = 525 nm. All the polystyrene microsphere suspensions were centrifuged, washed and redispersed into Milli-Q water 4 times before use. Graphene monolayer on copper foil (300 mm × 245 mm, monolayer coverage >99%) were manufactured by Chongqing Graphene Technology Company through the CVD method (Figure S1). Magnesium chloride (MgCl_2_), sodium chloride (NaCl) and calcium chloride (CaCl_2_) of AR grade were provided by Sinopharm Chemical Reagent Company. Polyethylene terephthalate (PET) films were produced by Shanghai Zicheng Packaging Materials Company. All the substrates and the salts were used as received.

### 3.2. Experimental Setup for Droplet Deposition and Drying

0.24 g of MgCl_2_ was dissolved into 10 mL Milli-Q water to prepare the aqueous solution at a concentration of 0.25 M. Then, twice echelon dilution was used to get a serial of concentrations.

To prepare the suspension containing mono-dispersed F-PS particles, 100 μL of F-PS particle suspension with single particle diameters (1.0 μm or 0.1 μm), 100 μL of Milli-Q water and 200 μL of salt solution with different concentrations were added into an Eppendorf tube, reciprocatingly sucked with a micropipette (Eppendorf), and ultra-sounded for 5 min to thoroughly mix them.

To prepare the suspension containing bi-dispersed F-PS particles, 100 μL of 1.0 μm diameter red F-PS particle suspension, 100 μL of 0.1 μm (or 0.2 μm) diameter green F-PS particle suspension and 200 μL of salt solution with different concentrations were added into an Eppendorf tube, reciprocatingly sucked with a micropipette (Eppendorf), and ultra-sounded for 5 min to thoroughly mix them.

Small droplets (0.2~1.5 μL) of the as-prepared suspensions containing mono/bi-dispersed F-PS particles and different concentrations of MgCl_2_ were then immediately deposited onto the substrate in a petri dish, which was loosely covered to avoid air disturbance or contaminants from the environment. The environment temperature was 18 ± 3.5 °C and the relative humidity was 47 ± 3.0%. The deposition of suspension droplets containing CaCl_2_ and NaCl followed the same procedure.

### 3.3. Measurement of the Separation Rate

The separation rate between F-PS particles with diameters of 1.0 μm and 0.1 μm, which is denoted by the mass ratio, was measured using a dynamic laser nanoparticle sizer (Zetasizer Nano ZS90, Malvern, UK). About 35 independently dried deposits from suspensions containing bi-dispersed F-PS particles and 2.0 mM MgCl_2_ on PET substrate were used for every single measurement. The rim part (about 1/4 of the outermost part along the radius, see Figure 1g) of each dried deposit was removed using tape and a long needle with a small hook on the tip. The remaining central parts of the independent dried deposits, together with the small pieces of PET substrates were placed into an Eppendorf tube with 2.0 mL Milli-Q water. The mixture was ultra-sounded for 30 min to redisperse the F-PS particles into the suspension. Then the small pieces of PET substrates were removed and the suspensions were used for the measurement of the mass ratio. Considering that the dynamic laser nanoparticle sizer demands a high particle concentration (10 mg/mL), we re-prepared the bi-dispersed suspension for separation in the second and third separating cycles, according to the mass ratio of particles with different sizes measured in the previous cycle. In other words, the concentration of particles with a diameter of 1.0 μm was maintained as 0.25% *w*/*v* in all bi-dispersed suspensions for separation, while the concentrations of particles with a diameter of 0.1 μm were 0.25, 0.076 and 0.033% *w*/*v* for the first, second and third separating cycles, respectively.

### 3.4. Imaging of the Deposits

Morphologies of the deposit patterns were captured by scanning electron microscopy (LEO 1530VP, Zeiss, Oberkochen, Germany). Fluorescence images of the deposit patterns were captured by optical fluorescence microscopy (BX51, Olympus, Tokyo, Japan). All images were acquired in similar illumination conditions and acquisition settings. They are displayed without any post-processing.

### 3.5. Contact Angle of the Graphene and PET Substrates

Droplets (~0.2 μL) of suspensions containing bi-dispersed F-PS particles (1.0 μm and 0.1 μm in diameters) and different concentrations of MgCl_2_ (0 mM and 6.0 mM) were placed on single-layer graphene and PET substrates. The contact angles were immediately examined by a surface tensiometer (Attension Theta, Biolin, Gothenburg, Sweden). The results are shown in Figure S6, which demonstrates that both the single-layer graphene and the PET substrates have contact angles larger than 75°.

## 4. Conclusions

In summary, we experimentally achieved the spontaneous separation of fluorescent polystyrene particles of different sizes in small-volume specimens by only adding trace amounts of cations (i.e., Mg^2+^, Ca^2+^ or Na^+^). This cation-controlled separation method enlarges the separation distance between particles of different sizes in the deposit pattern (e.g., at a millimeter level for a specimen volume as low as 0.2 μL), which is convenient for subsequent processing such as manually sampling from the deposit pattern and performing multiple separating cycles. The mass ratios between F-PS particles with diameters of 1.0 μm and 0.1 μm were effectively increased from 1.0 ± 0.1 to 12.9 ± 2.2 by merely three separating cycles. A theoretical analysis indicates that this cation-controlled spontaneous separation is attributed to the size-dependent adsorption of particles onto the aromatic substrate due to the strong hydrated cation-π interactions. We note that innovative morphological analysis tools have recently been developed by Lotito et al. [48,49,50,51] which would provide quantitative information, such as the spatial configurations of 2D assembly patterns, and cast light on understanding the dynamics of particle separation in the future. Overall, our findings provide a simple, maneuverable and low-cost method of achieving size-dependent micro/nanoparticles spontaneous separation of small-volume specimens, taking a step forward to the miniaturizing and automating of sample preparation processes. It will benefit a wide range of applications involving purification, bioassay, clinical diagnosis, chemical analysis and lab-on-a-chip devices.

## Data Availability

Not applicable.

## Supplementary Materials

The following supporting information can be downloaded at: https://www.mdpi.com/article/10.3390/ijms23158055/s1.

## References

[1] Hatti-Kaul R., Mattiasson B. (2003). Isolation and Purification of Proteins.

[2] Saxena A., Tripathi B.P., Kumar M., Shahi V.K. (2009). Membrane-based techniques for the separation and purification of proteins: An overview. Adv. Colloid Interface Sci..

[3] Haukanes B.I., Kvam C. (1993). Application of Magnetic Beads in Bioassays. Nat. Biotechnol..

[4] Hempen C., Karst U. (2006). Labeling strategies for bioassays. Anal. Bioanal. Chem..

[5] Gómez-Hens A., Fernández-Romero J., Aguilar-Caballos M. (2008). Nanostructures as analytical tools in bioassays. TrAC Trends Anal. Chem..

[6] Yager P., Edwards T., Fu E., Helton K., Nelson K., Tam M.R., Weigl B.H. (2006). Microfluidic diagnostic technologies for global public health. Nature.

[7] Marrazza G., Chianella I., Mascini M. (1999). Disposable DNA electrochemical sensor for hybridization detection. Biosens. Bioelectron..

[8] Whitesides G.M. (2006). The origins and the future of microfluidics. Nature.

[9] Ho C.-M., Tai Y.-C. (1998). Micro-electro-mechanical-systems (MEMS) and fluid flows. Annu. Rev. Fluid Mech..

[10] Garcia-Cordero J.L., Fan Z.H. (2017). Sessile droplets for chemical and biological assays. Lab Chip.

[11] Lin S.-C., Lu J.-C., Sung Y.-L., Lin C.-T., Tung Y.-C. (2013). A low sample volume particle separation device with electrokinetic pumping based on circular travelling-wave electroosmosis. Lab Chip.

[12] Jiang J., Mu L., Qiang Y., Yang Y., Wang Z., Yi R., Qiu Y., Chen L., Yan L., Fang H. (2021). Unexpected Selective Absorption of Lithium in Thermally Reduced Graphene Oxide Membranes. Chin. Phys. Lett..

[13] Lee H., Kang S.J., Lee J., Park K.H., Rhee W.J. (2022). Isolation and Characterization of Urinary Extracellular Vesicles from Healthy Donors and Patients with Castration-Resistant Prostate Cancer. Int. J. Mol. Sci..

[14] Destgeer G., Jung J.H., Park J., Ahmed H., Sung H.J. (2016). Particle Separation inside a Sessile Droplet with Variable Contact Angle Using Surface Acoustic Waves. Anal. Chem..

[15] Shi J., Huang H., Stratton Z., Huang Y., Huang T.J. (2009). Continuous particle separation in a microfluidic channel via standing surface acoustic waves (SSAW). Lab Chip.

[16] Akther A., Walsh E.P., Reineck P., Gibson B.C., Ohshima T., Abe H., McColl G., Jenkins N.L., Hall L.T., Simpson D.A. (2021). Acoustomicrofluidic Concentration and Signal Enhancement of Fluorescent Nanodiamond Sensors. Anal. Chem..

[17] Trantum J.R., Wright D.W., Haselton F.R. (2011). Biomarker-Mediated Disruption of Coffee-Ring Formation as a Low Resource Diagnostic Indicator. Langmuir.

[18] Liang L., Zhang C., Xuan X. (2013). Enhanced separation of magnetic and diamagnetic particles in a dilute ferrofluid. Appl. Phys. Lett..

[19] Saroj S.K., Panigrahi P.K. (2021). Magnetophoretic Control of Diamagnetic Particles Inside an Evaporating Droplet. Langmuir.

[20] Ilosvai A.M., Dojcsak D., Váradi C., Nagy M., Kristály F., Fiser B., Viskolcz B., Vanyorek L. (2022). Sonochemical Combined Synthesis of Nickel Ferrite and Cobalt Ferrite Magnetic Nanoparticles and Their Application in Glycan Analysis. Int. J. Mol. Sci..

[21] Krasitskaya V.V., Kudryavtsev A.N., Yaroslavtsev R.N., Velikanov D.A., Bayukov O.A., Gerasimova Y.V., Stolyar S.V., Frank L.A. (2022). Starch-Coated Magnetic Iron Oxide Nanoparticles for Affinity Purification of Recombinant Proteins. Int. J. Mol. Sci..

[22] Gascoyne P.R., Vykoukal J. (2002). Particle separation by dielectrophoresis. Electrophoresis.

[23] Gagnon Z.R. (2011). Cellular dielectrophoresis: Applications to the characterization, manipulation, separation and patterning of cells. Electrophoresis.

[24] McGrath J., Jimenez M., Bridle H. (2014). Deterministic lateral displacement for particle separation: A review. Lab Chip.

[25] Sajeesh P., Sen A.K. (2013). Particle separation and sorting in microfluidic devices: A review. Microfluid. Nanofluidics.

[26] Senyuk B., Liu Q., He S., Kamien R.D., Kusner R.B., Lubensky T.C., Smalyukh I.I. (2013). Topological colloids. Nature.

[27] Urdea M., Penny L.A., Olmsted S.S., Giovanni M.Y., Kaspar P., Shepherd A., Wilson P., Dahl C.A., Buchsbaum S., Moeller G. (2006). Requirements for high impact diagnostics in the developing world. Nature.

[28] Deegan R.D., Bakajin O., Dupont T.F., Huber G., Nagel S.R., Witten T.A. (1997). Capillary flow as the cause of ring stains from dried liquid drops. Nature.

[29] Deegan R., Bakajin O., Dupont T.F., Huber G., Nagel S.R., Witten T.A. (2000). Contact line deposits in an evaporating drop. Phys. Rev. E.

[30] Hu H., Larson R.G. (2005). Analysis of the Effects of Marangoni Stresses on the Microflow in an Evaporating Sessile Droplet. Langmuir.

[31] Deegan R.D. (2000). Pattern formation in drying drops. Phys. Rev. E.

[32] Wong T.-S., Chen T.-H., Shen X., Ho C.-M. (2011). Nanochromatography Driven by the Coffee Ring Effect. Anal. Chem..

[33] Monteux C., Lequeux F. (2011). Packing and sorting colloids at the contact line of a drying drop. Langmuir.

[34] Iqbal R., Majhy B., Shen A.Q., Sen A.K. (2018). Evaporation and morphological patterns of bi-dispersed colloidal droplets on hydrophilic and hydrophobic surfaces. Soft Matter.

[35] Devlin N.R., Loehr K., Harris M.T. (2015). The separation of two different sized particles in an evaporating droplet. AIChE J..

[36] Jeong H., Han C., Cho S., Gianchandani Y., Park J. (2018). Analysis of Extracellular Vesicles Using Coffee Ring. ACS Appl. Mater. Interfaces.

[37] Jeong H., van Tiem J., Gianchandani Y., Park J. (2014). Nanoparticle Separation Using Marangoni Flow in Evaporating Droplets. Proceedings of the Solid-State Sensors.

[38] Yi J., Jeong H., Park J. (2018). Modulation of nanoparticle separation by initial contact angle in coffee ring effect. Micro Nano Syst. Lett..

[39] Bansal L., Seth P., Murugappan B., Basu S. (2018). Suppression of coffee ring: (Particle) size matters. Appl. Phys. Lett..

[40] Haijun Y., Yizhou Y., Shiqi S., Binghai W., Nan S., Xing L., Rongzheng W., Long Y., Zhengchi H., Xiaoling L. (2020). Controlling the Coffee Ring Effect on Graphene and Polymer by Cations. Chin. Phys. Lett..

[41] Yang Y., Liang S., Wu H., Shi G., Fang H. (2022). Revisit the Hydrated Cation-π Interaction at the Interface: A New View of Dynamics and Statistics. Langmuir.

[42] Shi G., Dang Y., Pan T., Liu X., Liu H., Li S., Zhang L., Zhao H., Li S., Han J. (2016). Unexpectedly Enhanced Solubility of Aromatic Amino Acids and Peptides in an Aqueous Solution of Divalent Transition-Metal Cations. Phys. Rev. Lett..

[43] Chen L., Shi G., Shen J., Peng B., Zhang B., Wang Y., Bian F., Wang J., Li D., Qian Z. (2017). Ion sieving in graphene oxide membranes via cationic control of interlayer spacing. Nature.

[44] Shi G., Chen L., Yang Y., Li D., Qian Z., Liang S., Yan L., Li L.H., Wu M., Fang H. (2018). Two-dimensional Na–Cl crystals of unconventional stoichiometries on graphene surface from dilute solution at ambient conditions. Nat. Chem..

[45] Song Y., Zhan J., Li M., Zhao H., Shi G., Wu M., Fang H. (2022). Enhancement of the Water Affinity of Histidine by Zinc and Copper Ions. Int. J. Mol. Sci..

[46] Mahadevi A.S., Sastry G.N. (2013). Cation-π interaction: Its role and relevance in chemistry, biology, and material science. Chem. Rev..

[47] Lotito V., Zambelli T. (2017). Approaches to self-assembly of colloidal monolayers: A guide for nanotechnologists. Adv. Colloid Interface Sci..

[48] Lotito V., Zambelli T. (2016). Self-Assembly of Single-Sized and Binary Colloidal Particles at Air/Water Interface by Surface Confinement and Water Discharge. Langmuir.

[49] Lotito V., Zambelli T. (2018). Pattern Formation in Binary Colloidal Assemblies: Hidden Symmetries in a Kaleidoscope of Structures. Langmuir.

[50] Lotito V., Zambelli T. (2019). A Journey Through the Landscapes of Small Particles in Binary Colloidal Assemblies: Unveiling Structural Transitions from Isolated Particles to Clusters upon Variation in Composition. Nanomaterials.

[51] Lotito V., Zambelli T. (2020). Pattern detection in colloidal assembly: A mosaic of analysis techniques. Adv. Colloid Interface Sci..

