# Supplementary material for: Size-Dependent Spontaneous Separation of Colloidal Particles in Sub-Microliter Suspension by Cations
Source: Int J Mol Sci. 2022 Jul 22;23(15):8055. doi: 10.3390/ijms23158055 (PMC9329736; doi:10.3390/ijms23158055)
Supplement: Supplementary file 1 [file ijms-23-08055-s001.zip › ijms-1822445-supplementary.pdf]

Supplementary Information for

Size-Dependent Spontaneous Separation of Colloidal Particles in Sub-microliter Suspension by Cations

Context:

PS 1: A graphene substrate grown on copper foil by chemical vapor deposition (CVD)

PS 2: The distribution of F-PS particles with diameters of 1.0 μm and 0.1 μm along the outward radial direction within the deposit pattern

PS 3: Size-dependent separation of particles controlled by $MgCl_2$, $CaCl_2$ and NaCl with different concentrations

PS 4: Estimation of the dependence of the attractive force induced by hydrated cation-π interactions on the particle diameter

PS 5: Contact angles of suspension droplets on graphene and PET substrates

PS 1. A graphene substrate grown on copper foil by chemical vapor deposition (CVD)

Figure S1 shows a photograph of a graphene substrate grown on a copper foil by chemical vapor deposition (CVD), which is manufactured by Chongqing Graphene Technology Company. The coverage of graphene monolayer is more than 99%.

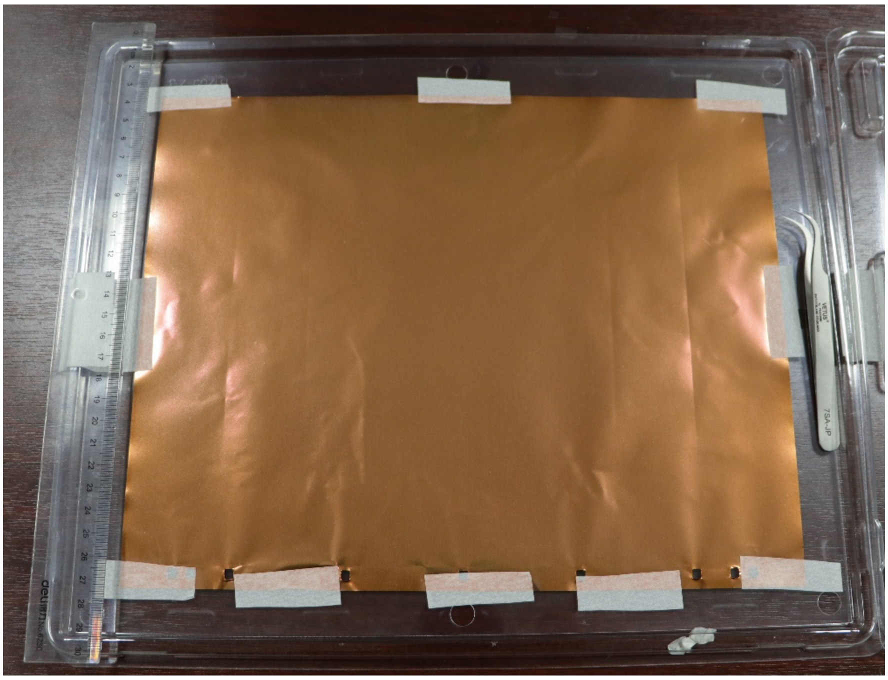

Figure S1. Photograph of a graphene substrate grown on copper foil by chemical vapor deposition (CVD).

PS 2. The distribution of F-PS particles with diameters of 1.0 μm and 0.1 μm along the outward radial direction within the deposit pattern.

Figure S2 shows the distribution of F-PS particles with diameters of 1.0 μm and 0.1 μm along the outward radial direction within the deposit pattern dried from suspension containing bi-dispersed F-PS particles and 2.0 mM $MgCl_2$. It is observed that the large particles uniformly distribute throughout the deposit pattern, while most of the small particles accumulate at the rim. The width of the rim part, where the majority of the small particles accumulate, is about only 1/5 of the radius of the deposit. This observation indicates that the separation distance between large and small particles is relatively large within the deposit.

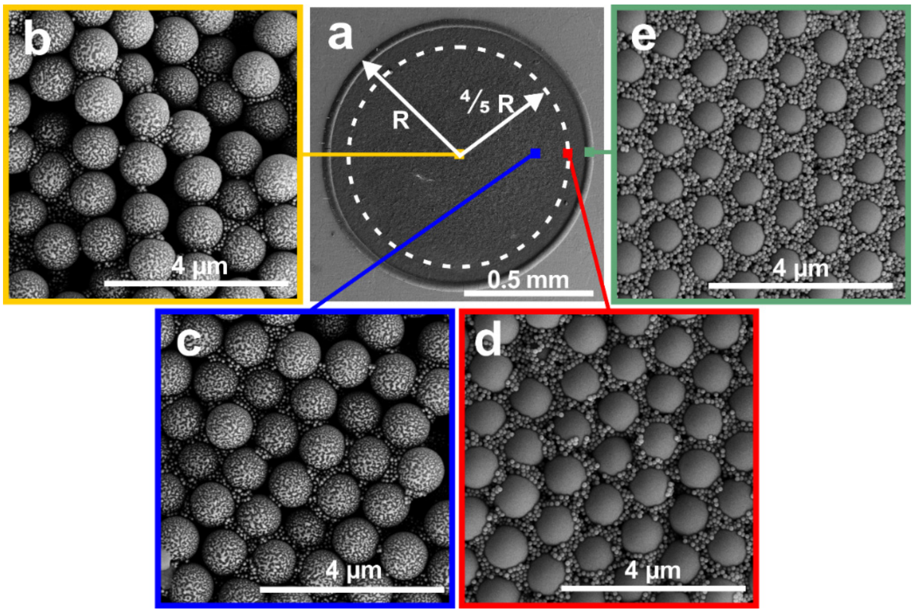

Figure S2. The distribution of F-PS particles with diameters of 1.0 μm and 0.1 μm along the outward radial direction within the deposit pattern. (a) SEM images of the deposit pattern dried from suspension containing bi-dispersed F-PS particles (1.0 μm and 0.1 μm in diameters) and 2.0 mM $MgCl_2$ on a single layer graphene. (b-e) The zoom-in SEM images of the selected areas along the outward radial direction.

PS 3. Size-dependent separation of particles controlled by $MgCl_2$, $CaCl_2$ and NaCl with different concentrations

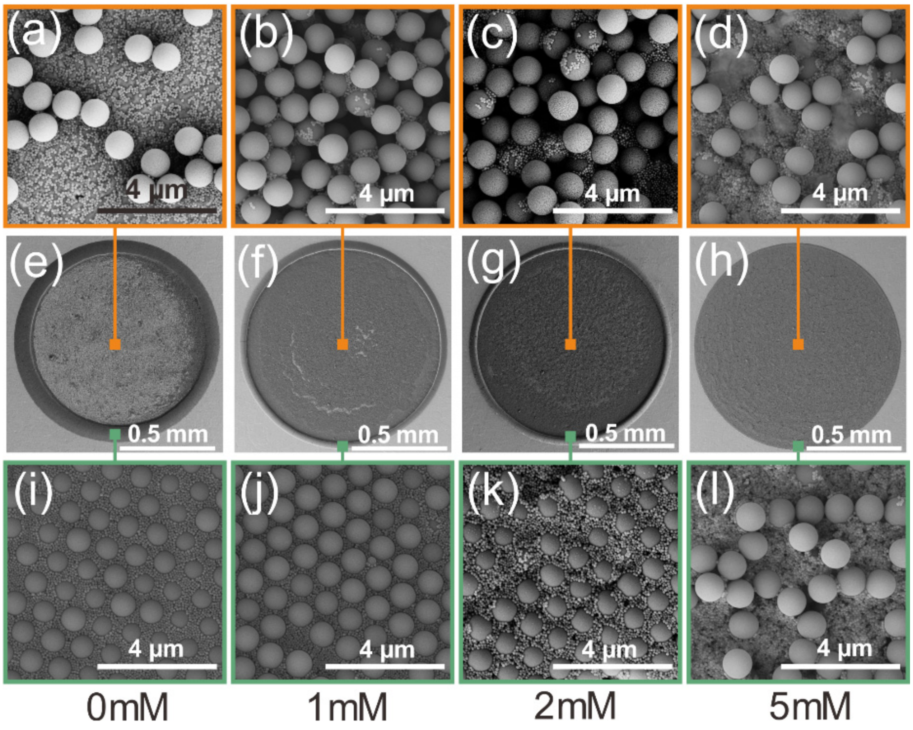

Figure S3. The size-dependent spontaneous separation between F-PS particles with diameters of 1.0 μm and 0.1 μm on a single layer graphene by adding trace amounts of

$MgCl_2$. (a-d) The zoom-in SEM images of the selected area at the center of the corresponding deposit patterns, respectively. (e-h) SEM images of deposit patterns on graphene dried from suspensions containing bi-dispersed F-PS particles and different concentrations of $MgCl_2$. (i-l) The zoom-in SEM images of the selected area at the rim of the corresponding deposit patterns, respectively.

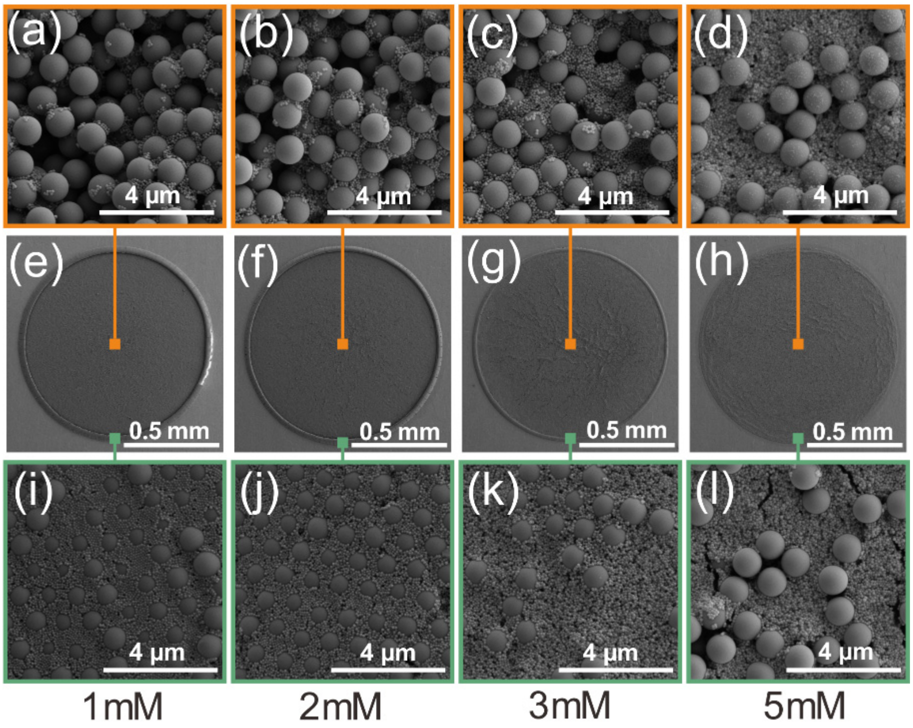

Figure S4. The size-dependent spontaneous separation between F-PS particles with diameters of 1.0 μm and 0.1 μm on a single layer graphene by adding trace amounts of $CaCl_2$. (a-d) The zoom-in SEM images of the selected area at the center of the corresponding deposit patterns, respectively. (e-h) SEM images of deposit patterns on graphene dried from suspensions containing bi-dispersed F-PS particles and different concentrations of $CaCl_2$. (i-l) The zoom-in SEM images of the selected area at the rim of the corresponding deposit patterns, respectively.

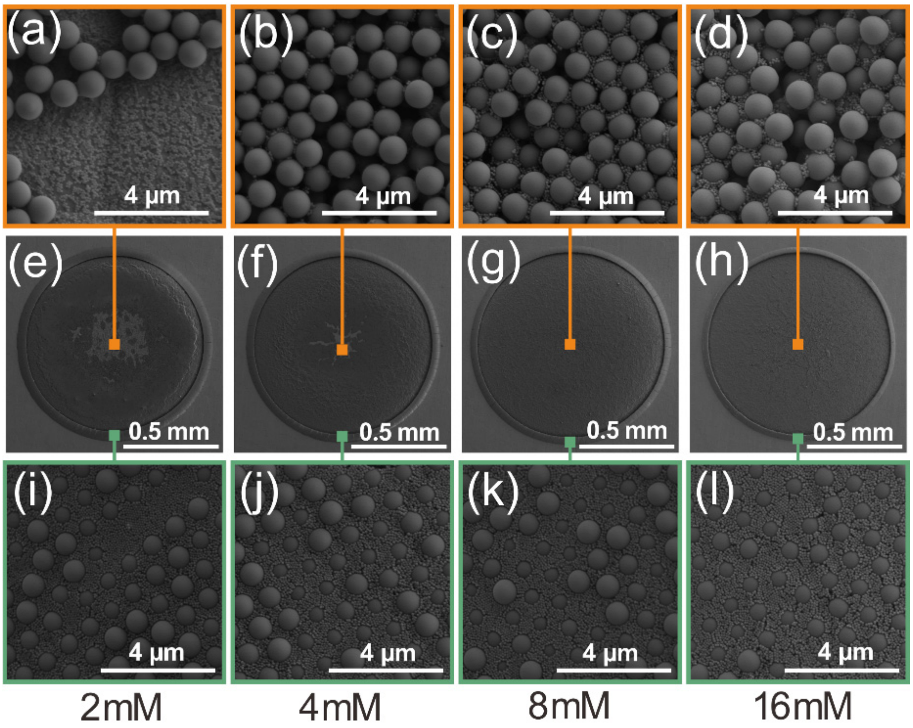

Figure S5. The size-dependent spontaneous separation between F-PS particles with diameters of 1.0 μm and 0.1 μm on a single layer graphene by adding trace amounts of NaCl. (a-d) The zoom-in SEM images of the selected area at the center of the corresponding deposit patterns, respectively. (e-h) SEM images of deposit patterns on graphene dried from suspensions containing bi-dispersed F-PS particles and different concentrations of NaCl. (i-l) The zoom-in SEM images of the selected area at the rim of the corresponding deposit patterns, respectively.

PS 4. Estimation of the dependence of the attractive force induced by hydrated cation-π interactions on the particle diameter

Due to the strong hydrated cation-π interactions between hydrated cations and aromatic rings, the adsorption probability of a cation onto an aromatic-rich surface is proportional to the surface area ($p \propto \alpha S$) when the surface is immersed in solutions with a given cation concentration, where α is the adsorption constant depending on the properties of the cation and surface as well as the cation concentration. When a F-PS particle with a dimeter of R approaches to the graphene surface driven by the random thermal motion, we could estimate the effective adsorption areas of the hydrated cation-π interactions on the graphene substrate $S_{\text{eff-graphene}}$ and particle surface $S_{\text{eff-ps}}$ (the surface of a spherical cup).

As shown in Figure 4b, the maximum interaction distance of the cation-mediated hydrated cation-π interactions between F-PS particle and the graphene substrate is denoted by r_{max}. As the interaction distance of hydrated cation-π interaction (less than 1 nm) is small relative to the particle diameter R, it is easy to verify that both the effective interaction areas $S_{\text{eff-ps}}$ and $S_{\text{eff-graphene}}$ are approximately proportional to the

particle diameter R. As a result, when a particle approaches to the graphene substrate driven by the random thermal motion, the probability that a cation simultaneously adsorbs to the graphene substrate and the F-PS particle surface could be expressed as

$$p_{adsorb} \propto \alpha_{ps} S_{eff-ps} \times \alpha_{graphene} S_{graphene} \propto R^2.$$

Thus, for a F-PS particle approaching to the graphene substrate, the attractive force F_A between the particle and substrate induced by the hydrated cation-π interactions, which is proportional to the adsorption probability P_{adsorb}, is approximately proportional to the squared particle diameter as $F_A \sim R^2$.

PS 5: Contact angles of suspension droplets on graphene and PET substrates

Figure S6 shows the contact angles of suspension droplets containing bi-dispersed F-PS particles (1.0 μm and 0.1 μm in diameters) and different concentrations of $MgCl_2$ (0 mM and 6.0 mM) on single-layer graphene and PET substrates. The results demonstrate that both the single-layer graphene and the PET substrates have contact angles larger than 75°

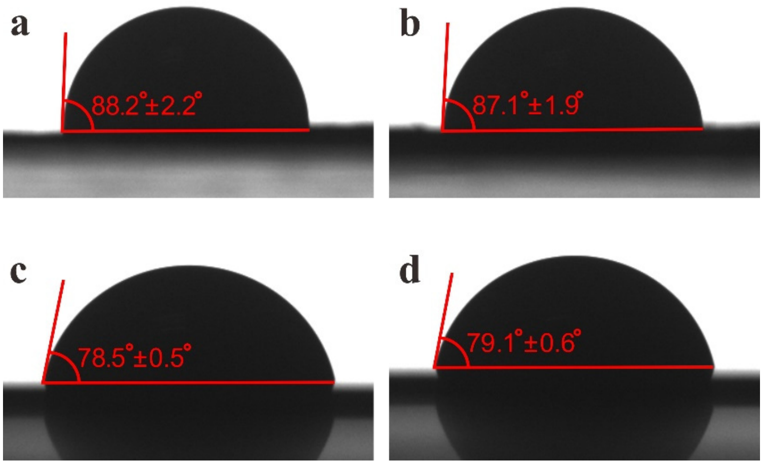

Figure S6. The contact angles of suspension droplets containing bi-dispersed F-PS particles (1.0 μm and 0.1 μm in diameters) and different concentrations of $MgCl_2$ (0 mM and 6.0 mM) on single-layer graphene and PET substrates. (a) 0 mM $MgCl_2$ on graphene substrate. (b) 6.0 mM $MgCl_2$ on graphene substrate. (c) 0 mM $MgCl_2$ on PET substrate. (d) 6.0 mM $MgCl_2$ on PET substrate.
